# Microbial modulation of proteoglycan and glycosaminoglycan biosynthesis in a three-dimensional corneal epithelium model

**DOI:** 10.3389/fcimb.2026.1804227

**Published:** 2026-05-28

**Authors:** Noelia Blanco-Agudín, Francisco Pérez-Rastrollo, Natalia Vázquez, Suhui Ye, Cristina Sánchez-Fernández, María Daniela Corte-Torres, Jesús Merayo-Lloves, Iván Fernández-Vega, Luis M. Quirós

**Affiliations:** 1 Instituto Universitario Fernández-Vega (IUFV), Fundación de Investigación Oftalmológica, Oviedo, Spain; 2 Departamento de Biología Funcional, University of Oviedo, Oviedo, Spain; 3 Instituto de Investigación Sanitaria del Principado de Asturias (ISPA), Oviedo, Spain; 4 Department of Pathology, Hospital Universitario Central de Asturias (HUCA), Oviedo, Spain; 5 Biobank of Principality of Asturias, Oviedo, Spain

**Keywords:** chondroitin sulfate, corneal epithelium, glycosaminoglycans, heparan sulfate, host–microbe interaction, ocular surface, proteoglycans, three-dimensional cell culture

## Abstract

**Background:**

Recently, a three-dimensional *in vitro* corneal epithelium model, designated QobuR, was developed. This model aims to reproduce the complex cellular interactions observed *in vivo* and to provide an alternative to animal models for toxicity testing and ophthalmic drug development. However, the corneal epithelium, like other exposed epithelia, is continuously exposed to microorganisms present on the ocular surface, although the existence and extent of a stable corneal microbiota remain areas of ongoing investigation. Nonetheless, microbial exposure may exert a significant influence on its physiology. During microbial interaction with epithelial surfaces, cell-surface proteoglycans and glycosaminoglycans act as key mediators, and this interaction can induce changes in their biosynthesis and structure. As these molecules function as specific receptors for numerous ligands and regulate essential cellular processes, their interaction with the microbiota may contribute to the maintenance of tissue homeostasis.

**Methods:**

In this study, we analyzed the transcriptional profiles of genes involved in PG and GAG biosynthesis in the QobuR model following controlled co-culture with individual bacterial species and with a mixed microbial consortium designed to represent the ocular surface microbiota. Expression changes were assessed by qRT-PCR and complemented by immunohistochemical analysis.

**Results:**

Distinct bacterial species induced specific patterns of gene expression, predominantly affecting enzymes involved in late-stage sulfation of heparan sulfate and chondroitin sulfate chains. Exposure to the microbial consortium resulted in broader and more complex transcriptional modulation, incorporating most changes observed in individual conditions while introducing additional alterations. Notably, the transcriptional profile of microbiota-exposed QobuR showed increased similarity to donor-derived corneal epithelium. These observations were supported by immunohistochemical analyses.

**Conclusions:**

These findings demonstrate that corneal epithelial cells exhibit dynamic transcriptional responses to microbial exposure under controlled *in vitro* conditions. The results highlight the relevance of incorporating host–microbe interaction components into three-dimensional epithelial models, while also emphasizing that the interpretation of these responses should consider the experimental framework and the current uncertainties regarding the composition and the functional role of the ocular surface microbiota.

## 1 Introduction

The cornea forms the anterior part of the eye and is composed of a highly specialized tissue that allows light transmission. It also accounts for two-thirds of the eye’s refractive power (Knupp et al., 2009). The corneal epithelium (CE) constitutes the outermost layer of the cornea, representing approximately 10% of its thickness. It is composed of 5 to 7 layers, including superficial squamous cells, wing cells, and basal cells. The CE performs several essential functions for ocular physiology, including hydration, nutrition, protection, and maintaining corneal transparency (Abtahi et al., 2024).

The structure of the CE has been modeled through the development of three-dimensional *in vitro* systems. These models aim to reproduce the complex cellular interactions observed *in vivo* and their influence on proliferation, differentiation, and cell survival (Baker and Chen, 2012; Bonnier et al., 2015). In addition to serving as a tool for the study of corneal physiology, regeneration, and pathology, CE models are increasingly being used as technological platforms for toxicity testing, providing an alternative to animal models, as well as for ophthalmic drug development (Kaluzhny and Klausner, 2021).

Due to its exposure to the external environment, the CE, like other exposed epithelia, is in continuous contact with microorganisms present on the ocular surface, although the presence of a stable and resident microbiota at the corneal level remains a subject of active debate (Herzog et al., 2023; Zilliox and Bouchard, 2023; Kaur et al., 2025). This microbial presence does not typically elicit an inflammatory response in healthy individuals, whereas the CE can respond to ocular pathogens by producing pro-inflammatory cytokines (Petrillo et al., 2020). The ocular surface microbiota has been proposed to play roles analogous to those described in other epithelial tissues, although its composition, stability, and functional impact—particularly at the corneal level—are not yet fully established (Cavuoto et al., 2019).

During microbial interaction with epithelial surfaces, cell surface proteoglycans (PGs) and their glycosaminoglycan (GAG) chains are involved (Martín et al., 2013; Rajas et al., 2017; Martín et al., 2022). In the cornea, these molecules participate in the binding of both Gram-positive and Gram-negative bacteria in a differential manner, with heparan sulfate (HS) playing a central role (García et al., 2016b).

GAGs are linear polysaccharides composed of repeating disaccharide units. There are four main types, primarily differentiated by the structure of their disaccharide units and the degree and pattern of chemical modifications, especially sulfation, of their chains. Three of these families are found as components of PGs: HS, chondroitin sulfate (CS), and keratan sulfate. The fourth, hyaluronic acid, exists in free form (Ricard-Blum et al., 2024).

PGs are primarily located at the cell surface/pericellular region and within the extracellular matrix (ECM), though some may also be found intracellularly. HSPGs are predominantly localized at the cell surface, whereas CS- and keratan sulfate-containing PGs are more abundant in the ECM (Iozzo and Schaefer, 2015). The CE is a highly cellular tissue, although it also includes a basement membrane and pericellular matrix (Sridhar, 2018). Consequently, HSPGs are the most functionally relevant PGs in this tissue, though many are hybrid PGs that also contain CS chains (Iozzo and Schaefer, 2015). Hyaluronic acid has also been detected in the CE (Lin et al., 2022).

The biological functions of PGs depend largely on their GAG chains, although the core protein can influence GAG chain function (Lindahl, 2014). The structure of GAG chains in HS- and CS-PGs appears to be largely independent of the core protein and is primarily defined by the specific arrangement of sulfate groups, which creates distinct binding sites for numerous ligands. These ligands may exhibit high affinity for a specific GAG type (most notably HS) or bind to several types (Vallet et al., 2022). Such ligands include cytokines, chemokines, growth factors, enzymes and enzyme inhibitors, as well as ECM proteins (Whitelock and Iozzo, 2005). By modulating the binding of these ligands, PGs play a significant role in regulating essential cellular functions and maintaining tissue homeostasis (Ricard-Blum et al., 2024; Iozzo and Schaefer, 2015; Whitelock and Iozzo, 2005).

The interaction of microorganisms with HS and CS chains on cell surface PGs can induce changes in the transcription of enzymes responsible for GAG chain biosynthesis and structure (Martín et al., 2020; Ordiales et al., 2022). As ligand interactions and the resulting effects on cell physiology are mediated by these structures, interaction with the microbiota may be necessary for appropriate cellular homeostasis. However, current *in vitro* CE models are developed in the absence of commensal microorganisms. Recently, a novel human *in vitro* corneal epithelial model (QobuR), derived from normal limbal tissue, has been developed for applications including preclinical drug screening and ocular risk assessment (Chacón et al., 2019, 2022). This model consists of normal human corneal epithelial cells cultured at the air-liquid interface, forming a stratified squamous epithelium that recapitulates the morphological microstructure, barrier properties, and expression of specific biomarkers of native human CE (Chacón et al., 2019). Nevertheless, only limited data are available regarding PG and GAG expression in corneal epithelial models. Recent studies using corneal organoid systems derived from induced pluripotent stem cells, which include both epithelial and stromal components, have described the presence and distribution of glycosaminoglycans and proteoglycans, as well as the expression of related biosynthetic enzymes (Ashworth et al., 2025). However, these models resemble developing rather than mature corneal tissue and do not specifically address the regulation of PG and GAG biosynthesis in reconstructed epithelial systems. In this context, the modulation of these pathways under controlled microbial exposure remains largely unexplored.

In this study, we analyzed the transcriptional profiles of all key genes involved in PG and GAG biosynthesis in the QobuR model and examined how exposure to different bacterial species commonly found in the ocular microbiota affects this expression. We further developed an *in vitro* ocular microbiota model and compared its effects with those induced by individual bacterial species. We found that different bacterial species differentially modulate gene expression, while the mixed microbiota model produced a more extensive effect, incorporating most of the alterations observed with individual species along with additional changes, suggesting a complex synergistic effect. The most prominent alterations affected the fine sulfation pattern of HS and CS chains, particularly late-stage modifications. Comparing gene expression under the different experimental conditions to that of ex vivo human CE revealed that the expression profile of QobuR exposed to the microbiota model was more similar to the donor corneal epithelium than any other condition, including the axenic 3D model. Finally, immunohistochemical analysis of selected expression changes induced by microbiota exposure in the CE model further confirmed a marked increase in similarity to donor-derived corneal epithelia.

These findings underscore the importance of considering controlled microbial exposure and host–microbe interactions in the design of three-dimensional epithelial systems for research, toxicology, and pharmacological development.

It is important to note that, given the paucibacterial nature of the ocular surface and the technical limitations associated with its study, particularly at the corneal level, the existence, composition, and functional relevance of a corneal-associated microbiota remain areas of active investigation. In this context, experimental models based on controlled exposure of epithelial systems to representative microorganisms provide a useful framework to explore host–microbe interactions under defined conditions.

## 2 Materials and methods

### 2.1 Microorganisms and culture media

The bacterial species used in this study included *Pseudomonas aeruginosa*, *Corynebacterium* sp., *Propionibacterium* sp., *Acinetobacter baumannii*, *Staphylococcus epidermidis*, *Staphylococcus aureus*, *Streptococcus pneumoniae*, and *Streptococcus pyogenes*, all of which were clinical isolates from the Central University Hospital of Asturias (Oviedo, Spain). Bacteria were grown in Brain Heart Infusion Broth (BHI) (Merck, Sigma-Aldrich, St. Louis, MO, USA) at 37 °C with shaking, except for *S. pneumoniae*, which was cultured in a 5% (v/v) CO_2_ atmosphere without agitation, and the microaerophilic/aerotolerant *Propionibacterium* sp., which, to promote optimal growth, was cultured under anaerobic conditions using the AnaeroGen™ system (Thermo Scientific, Waltham, MA, USA) according to the manufacturer’s instructions.

### 2.2 Development of corneal epithelium models

Human donor corneal tissues were handled in accordance with the Declaration of Helsinki. To develop CE models, three human corneas were obtained from the Fernández-Vega Ophthalmology Institute (Oviedo, Asturias, Spain) from corneas previously used for penetrating keratoplasty. All tissues were stored at 4 °C in Eusol-C medium (Alchimia, Ponte S. Nicolò, Italy) for no more than 10 days prior to experimentation.

Model construction followed a previously described protocol (Chacón et al., 2019). Briefly, limbal tissues were dissected under a stereomicroscope, cut into small fragments, and digested with 0.25% trypsin/EDTA (Sigma-Aldrich, MO, USA) for 90 min at 37 °C. The resulting cell suspension was centrifuged (Eppendorf 5702R, Eppendorf, Hamburg, Germany) at 0.4 rcf for 10 min, and the supernatant was discarded. Cells were resuspended in fresh medium and seeded into 25 cm² culture plates. Once confluent, cells were digested with Accutase (Sigma-Aldrich, MO, USA), centrifuged, and reseeded onto ten 1.12 cm² Transwell^®^ inserts (0.4 µm pore size; Corning, NY, USA) in 12-well plates (50,000 cells/insert). Cultures were maintained with medium changes three times per week. For 3D differentiation, the culture medium was replaced with CnT-PR-3D (CellnTec Advanced Cell Systems AG; Bern, Switzerland), and the QobuR model was generated by culturing confluent human limbal epithelial cells at the air–liquid interface for 7 days.

### 2.3 Extraction of corneal epithelium from donor corneas

Three human corneas discarded by the Community Center for Blood and Tissue (Oviedo, Asturias, Spain) due to low endothelial cell density were used to isolate corneal epithelium. The corneal surface was gently scraped from the center to the periphery with a fine needle, and the epithelium was collected and transferred to tubes containing sterile PBS. All tissues were handled in accordance with the principles of the Declaration of Helsinki.

### 2.4 Bacterial co-culture with corneal epithelial model

Depending on the experiment, the CE models were exposed to microorganisms under controlled conditions for 15 h, allowing the assessment of epithelial responses to sustained host–microbe interaction rather than early adhesion events. Microorganisms were cultured to exponential phase (OD_600_ = 0.5) and added in 500 µL of PBS at a multiplicity of infection of 25 bacteria per epithelial cell. To prevent bacterial overgrowth during incubation, treatments were carried out in the presence of gentamicin at growth-inhibitory concentrations: 28 µM for *P. aeruginosa*, 15 µM for *S. epidermidis*, 75 µM for *Corynebacterium* sp., 113 µM for *S. aureus*, and 150 µM for *S. pyogenes*. For *S. pneumoniae*, erythromycin was used at 114 µM.

To simulate ocular microbiota, a mixed suspension containing 25 bacteria per epithelial cell was prepared with the following species proportions: *P. aeruginosa* (28%), *Propionibacterium* sp. (28%), *Corynebacterium* sp. (21%), *A. baumannii* (16.5%), *S. aureus* (2.5%), *S. epidermidis* (2.5%), *S. pyogenes* (1%), and *S. pneumoniae* (1%). Co-culture was carried out in the presence of 50 µM gentamicin.

### 2.5 Characterization of barrier function: trans-epithelial electrical resistance

TEER measurements were performed as previously described (Chacón et al., 2019), using the following equation:


TEER = (R_sample − R_blank) × effective area

where *R_sample* represents the resistance value of the cell culture models, *R_blank* corresponds to the resistance value of inserts without cultured cells, and the effective area was 1.12 cm².

### 2.6 RNA isolation, cDNA synthesis, and qRT-PCR

Total RNA was isolated using the RNeasy Kit (Qiagen; Hilden, Germany) following the manufacturer’s instructions. CE models were first washed three times with 500 µL PBS, and cells were detached by pipetting 200 µL/well of RLT buffer. For donor-derived corneal epithelia, samples were resuspended in 600 µL RLT buffer and homogenized using a Polytron PT 2100 (Kinematica Inc.; Bohemia, NY, USA) for 1 min at 30,000 rpm, followed by centrifugation at 15,000 × g to remove debris. To eliminate residual genomic DNA, samples were treated with RNase-Free DNase during the purification process (Qiagen). cDNA synthesis was performed using the High-Capacity cDNA Transcription Kit (Applied Biosystems; Foster City, CA, USA), as previously described (Fernández-Vega et al., 2015). qRT-PCR reactions and analysis of amplification products were carried out according to previously published protocols (Fernández-Vega et al., 2015). Primer sequences are listed in **Supplementary Table 1**. Glyceraldehyde-3-phosphate dehydrogenase (GAPDH) was used as a housekeeping gene for normalization of gene expression.

### 2.7 Immunohistochemistry

Immunohistochemical staining was performed using the EnVision™ FLEX High pH Kit and the Dako Autostainer System (Agilent Dako, Santa Clara, CA, USA). Paraffin-embedded tissue sections (3 µm) were deparaffinized, rehydrated, and subjected to heat-induced epitope retrieval at 95 °C for 20 min in EnVision™ FLEX Target Retrieval Solution, High pH 9.0 (Agilent Dako), using the Pre-Treatment Module PT-LINK (Agilent Dako). Endogenous peroxidase activity was quenched by incubation with EnVision™ FLEX Peroxidase-Blocking Reagent (Agilent Dako) for 5 min at room temperature. The following primary antibodies were used: Mouse monoclonal anti-HS epitope antibodies 10E4 and JM403 (1:100 dilution) from AMS Biotechnology (Abingdon, UK); Rabbit polyclonal anti-SPAM1 (1:100 dilution) and Rabbit polyclonal anti-HYAL1 (1:500 dilution) from Invitrogen (Waltham, MA, USA); Rabbit polyclonal anti-Heparanase-1 (1:50 dilution) from MilliporeSigma (Burlington, MA, USA); Mouse monoclonal anti-CS (clone CS-56; 1:100 dilution) from Sigma-Aldrich (St. Louis, MO, USA). Immunoreactivity was visualized using 3,3′-diaminobenzidine (DAB) as the chromogenic substrate, included in the EnVision™ FLEX/HRP Detection System (Agilent Dako). Sections were counterstained with hematoxylin, dehydrated through graded alcohols, cleared in xylene, and mounted with EnVision™ FLEX Mounting Medium (Agilent Dako). Negative controls were processed by omitting the primary antibody. Stained sections were examined and photographed under a bright-field light microscope.

### 2.8 Statistical analysis and graphical representation

The Mann–Whitney U test was used to compare the means of two different groups. For qPCR analyses, four CE models derived from independent donors were used, with each reaction performed in at least triplicate for each donor and additional replicates included when required. TEER measurements were carried out in eight control wells and eight microbiota-treated wells. Cluster analysis was performed using the Statistics for Windows software (StatSoft Inc.; Tulsa, OK, USA). Heatmaps were generated using R Statistical Computing. Venn diagrams were created with the nVenn tool (http://degradome.uniovi.es/cgi-bin/nVenn/nvenn.cgi) (Pérez-Silva et al., 2018), and UpSet plots were generated using the software available at https://www.chiplot.online/upset_plot.html.

## 3 Results

### 3.1 Each bacterial species generates a distinct pattern of gene expression alterations under the defined co-culture conditions used in this study

The transcriptional analysis of the 73 genes involved in GAG biosynthesis and editing included in this study detected mRNA expression for 65 of them in the QobuR model. Transcripts for only eight genes (*B3GAT2*, *EXTL1*, *HAS2*, *HAS3*, *NDST3*, *NDST4*, *HPSE2*, and *HYAL4*) were not detected (**Supplementary Figure 1**). Following exposure to different bacterial species, QobuR exhibited significant changes in the transcription of specific genes. These alterations followed species-specific patterns: while certain genes were broadly modulated, others responded exclusively to specific microbial species (**Supplementary Figure 1**).

**Figure 1:**
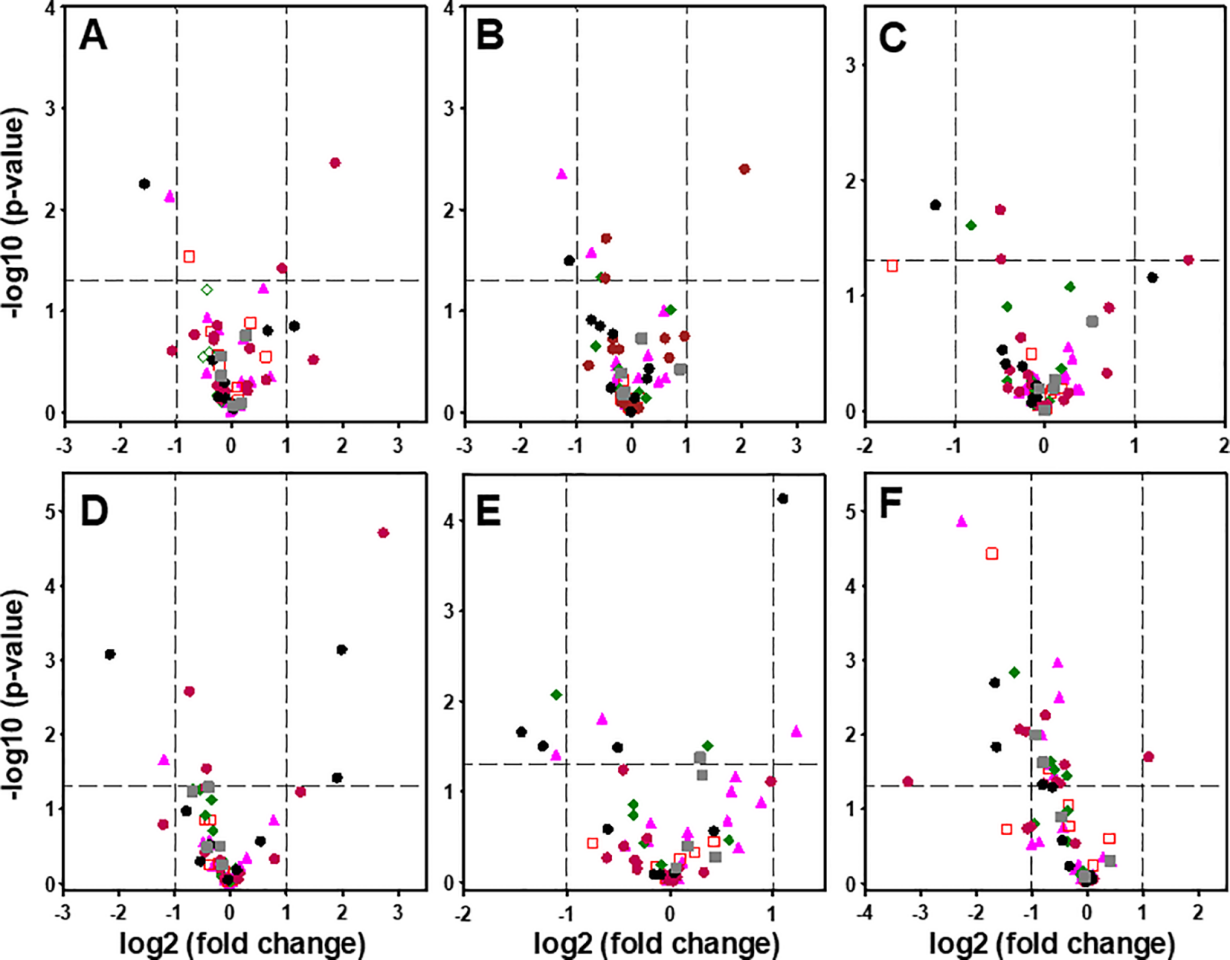
Volcano plots representing differential transcriptional analysis of PG- and GAG-coding genes induced by bacterial adherence to QobuR. The threshold in the volcano plot was p-value < 0.05 and |log_2_ fold change| > 1. **(A)** *Streptococcus pneumonia*; **(B)** *Streptococcus pyogenes*; **(C)** *Staphylococcus epidermidis*; **(D)** *Staphylococcus aureus*; **(E)** *Pseudomonas aeruginosa*; **(F)** *Corynebacterium* sp. Different gene groups are identified by distinct symbols: 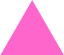, PGs; 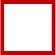, HS/CS tetrasaccharide linker; 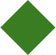, GAG glycosyltransferases; 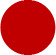, fine structure synthesis of HS; 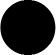, fine structure synthesis of CS; 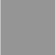, hydrolases.

Two *Streptococcus* species were used in this study: *S. pneumoniae* and *S. pyogenes*. Both induced very similar expression changes, affecting only three genes. The *SRGN* gene, encoding the PG serglycin, was downregulated by approximately 50% in both cases. Similarly, *CHST15*, which encodes an *N*-acetylgalactosamine 4-sulfate 6-*O*-sulfotransferase involved in CS biosynthesis, was also downregulated. Additionally, *HS3ST5*, which participates in C3 sulfation of the glucosamine residue in HS chains, was upregulated approximately fourfold in response to both microorganisms (**Figures 1A, B**).

Unlike the *Streptococcus* species, the two *Staphylococcus* species, *S. epidermidis* and *S. aureus*, induced distinct transcriptional changes in CE models. *S. epidermidis* triggered only an approximately threefold upregulation of *HS3ST5* and a >50% downregulation of *CHST15* (**Figure 1C**). In contrast, *S. aureus* induced a broader set of alterations, including an approximately 50% downregulation of *SRGN* and a sixfold upregulation of *HS3ST5*. Notably, the most affected genes were those involved in CS biosynthesis: *CHST13* and *CHST7* were upregulated approximately fourfold, whereas *CHST15* was downregulated by more than 75% (**Figure 1D**).

Exposure to *P. aeruginosa* led to significant modulation of six genes in QobuR, including *SDC4* (>2-fold upregulation) and *TGFBR3* (downregulated by approximately 50%). Again, genes related to CS biosynthesis were most affected: *CHSY3*, *CHST14*, and *CHST15* were downregulated by more than 50%, whereas *DSE* transcript levels doubled (**Figure 1E**).

The most pronounced transcriptional impact was observed upon exposure to *Corynebacterium* sp., which altered the expression of nine genes—eight downregulated and one upregulated. *SRGN* mRNA levels decreased by approximately 80%, and *B3GALT6* by about 70%. Three genes involved in CS biosynthesis (*CHSY3*, *CHST11*, and *CHST15*) showed reductions of 60–70%. Interestingly, HS biosynthesis was also strongly affected, with *HS6ST1* and *HS3ST3B1* downregulated by approximately 50%, *HS6ST2* by around 90%, and *HS3ST4* upregulated 2-fold (**Figure 1F**).

### 3.2 A bacterial ocular microbiota model induces widespread gene expression changes under controlled *in vitro* co-culture conditions

**Figure 2:**
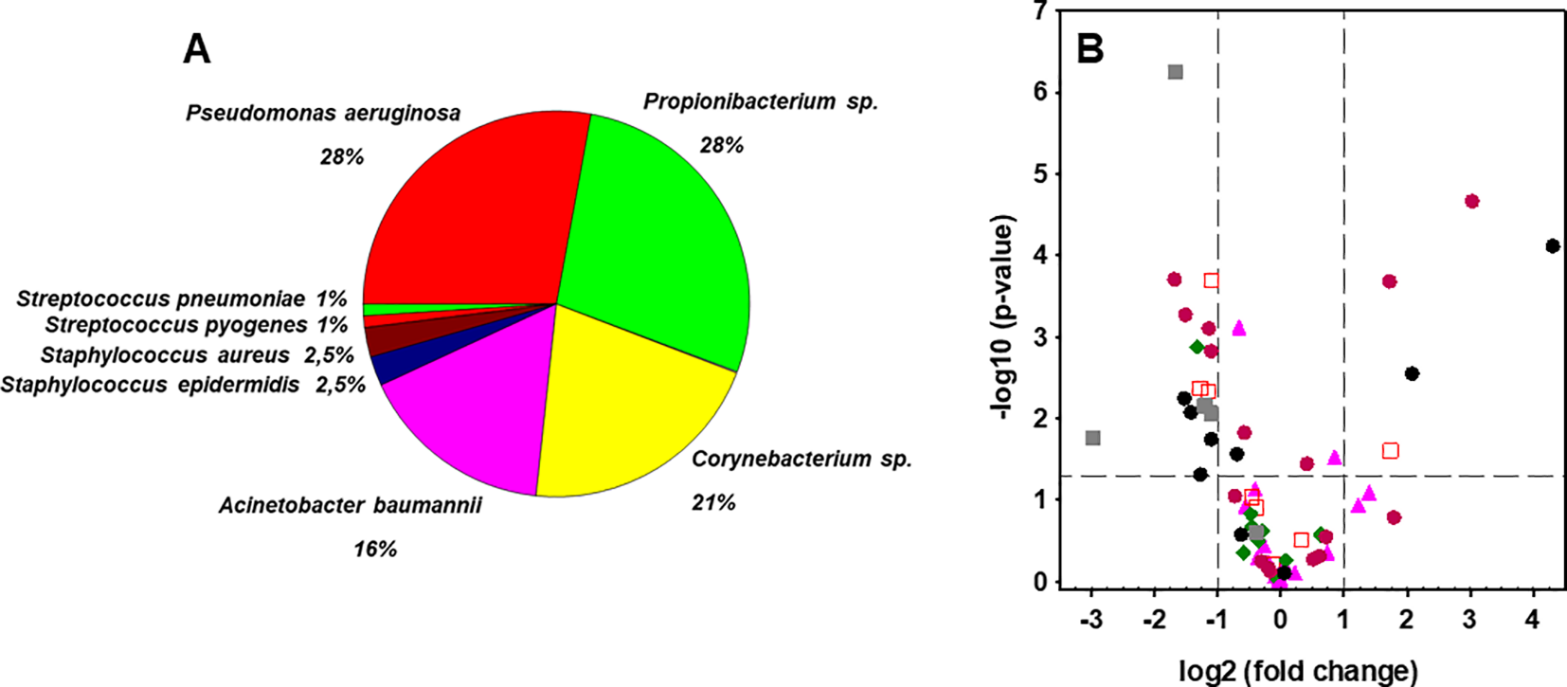
Differential transcriptional analysis of PG- and GAG-coding genes induced by adherence of an ocular microbiota model to QobuR. **(A)** specific composition and proportions of the ocular microbiota model. **(B)** Volcano plot representing differential transcription; the threshold in the volcano plot was p-value < 0.05 and |log_2_ fold change| > 1. Different gene groups are identified by distinct symbols: 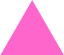, PGs; 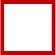, HS/CS tetrasaccharide linker; 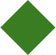, GAG glycosyltransferases; 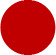, fine structure synthesis of HS; 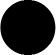, fine structure synthesis of CS; 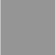, hydrolases.

The ocular surface is exposed to microbial communities collectively referred to as the ocular microbiota. Although a definitive core microbiome has not been established, current literature supports the presence of predominant genera in specific proportions (Okonkwo et al., 2020). Based on this, we developed a microbiota model including the genera *Pseudomonas*, *Propionibacterium*, *Corynebacterium*, *Acinetobacter*, *Staphylococcus*, and *Streptococcus*, with two species representing each of the latter two genera (**Figure 2A**). *A. baumannii* and *Propionibacterium* sp. were incorporated during the development of the mixed microbiota model and were therefore not included in the same individual downstream analyses performed for the initially evaluated bacterial species.

Differential gene expression analysis of QobuR upon exposure to this microbiota revealed a greater number of transcriptional changes than those induced by individual bacterial species (**Figure 2B**). No significant changes were observed in core PG protein-coding genes; however, several enzymes involved in GAG chain synthesis and modification were affected. Among genes encoding enzymes for the common HS/CS tetrasaccharide linker, *B3GALT6* was downregulated by approximately 50%, *B3GAT1* was upregulated, and both *FAM20B* and *PXYLP1* were downregulated to a similar extent.

HS chain biosynthesis was significantly altered, with *NDST1*, *NDST2*, *HS6ST1*, and *HS3ST6* downregulated by 50–70%, together with *HPSE* (an endoglycosidase), and upregulation of *HS3ST4* (>3-fold) and *HS3ST5* (>8-fold). CS biosynthesis was also markedly affected, with *CHSY3*, *CHST11*, *CHST14*, *CHST15*, and *CHST3* downregulated to below 50%, whereas *CHST13* was upregulated approximately fourfold and *CHST7* nearly 20-fold. Lastly, downregulation of the hyaluronic acid-degrading enzymes *HYAL1*, *HYAL2*, and *SPAM1* was observed (**Figure 2B**).

### 3.3 Expression changes affect a minority of genes and are focused on specific targets

**Figure 3:**
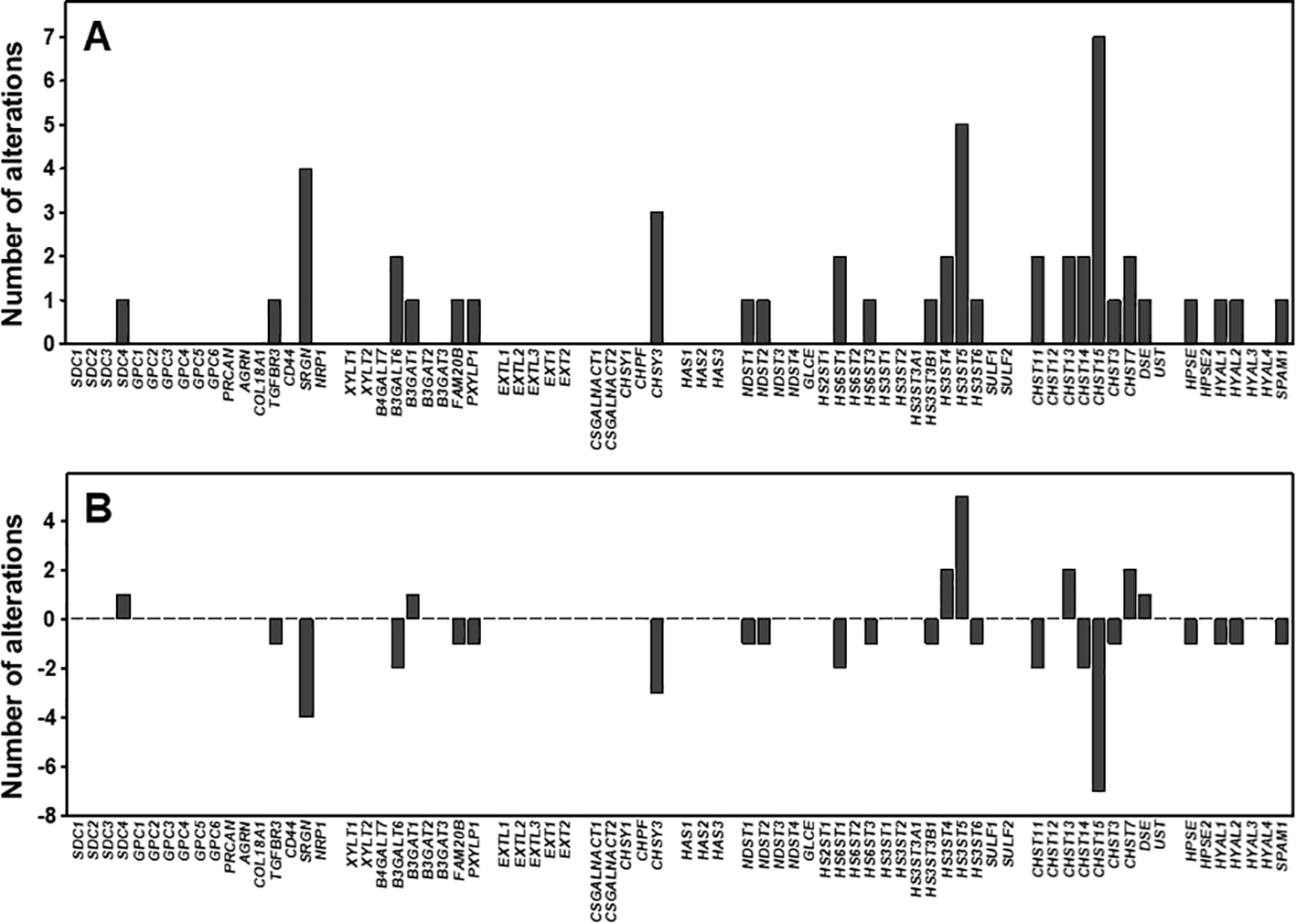
Number of expression changes detected for each analyzed gene. **(A)** Total changes; histogram bars indicate the number of experimental conditions, among all those tested, in which each gene showed altered expression. **(B)** Over- and underexpression; positive bars indicate the number of conditions in which each gene was overexpressed, whereas negative bars indicate the number of conditions in which it was underexpressed.

The transcription of 46 out of the 73 genes analyzed (63%) remained unchanged under all experimental conditions, whereas 27 genes (37%) showed deregulation. Of these, 16 genes were altered under a single condition and seven under two conditions. In contrast, *CHSY3* was altered in three conditions, *SRGN* and *HS3ST5* in four and five conditions, respectively, and *CHST15* in all seven experimental settings (**Figure 3A**).

Most of the dysregulated genes (20 in total) were downregulated relative to the axenic CE model, whereas seven were upregulated (**Figure 3B**). It is noteworthy that, for each gene in which transcriptional alterations were detected, the direction of the change in expression was conserved in all cases where it occurred. Thus, a specific gene could experience over- or under-expression, but no cases were observed where the type of alteration was dependent on the specific microorganism (**Figure 3B**).

**Figure 4:**
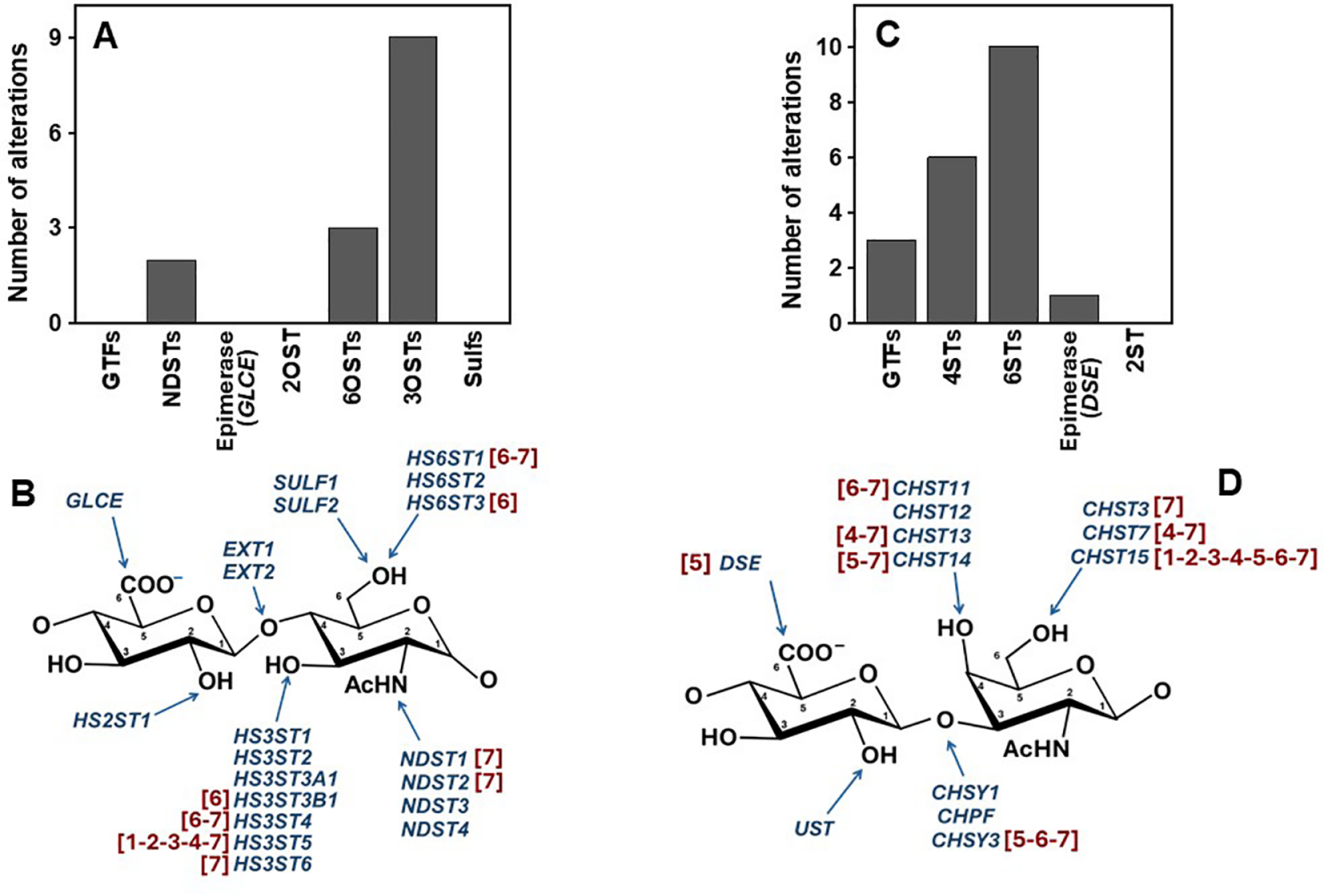
Transcriptional alterations observed in genes involved in HS and CS chain biosynthesis. **(A)** Total transcriptional changes in genes involved in each stage of HS chain biosynthesis. **(B)** HS disaccharide unit indicating the genes involved at each biosynthetic step, with treatment conditions showing significant alterations highlighted in red. **(C)** Total transcriptional changes in genes involved in each stage of CS chain biosynthesis. **(D)** CS disaccharide unit indicating the genes involved at each biosynthetic step, with treatment conditions showing significant alterations highlighted in red. Numbers indicate the different treatment conditions: 1, *S. pneumoniae*; 2, *S. pyogenes*; 3, *S. epidermidis*; 4, *S. aureus*; 5, *P. aeruginosa*; 6, *Corynebacterium* sp.; 7, microbiota.

Excluding SRGN, the most frequent transcriptional changes occurred in genes involved in HS (14 alterations) and CS (20 alterations) biosynthesis, representing 34% and 49% of the total, respectively. Together, these changes accounted for 83% of all observed alterations. Specifically, although changes in HS biosynthesis affected some genes encoding NDST and 6OST isoforms, the largest number of detected alterations involved transcripts of genes encoding enzymes responsible for C3 sulfation of glucosamine residues (**Figure 4A**). The microbiota model induced the most substantial changes, affecting transcripts of the two detected *NDST*, *HS6ST1*, and *HS3ST4*, *HS3ST5*, and *HS3ST6* (**Figure 4B**). Notably, *HS3ST5* was also overexpressed in response to all *Streptococcus* and *Staphylococcus* species tested (**Figure 4B**).

In CS biosynthesis, most alterations affected C4 and, particularly, C6 sulfation of galactosamine residues. *CHSY3* polymerase expression was also altered (**Figure 4C**). Again, the microbiota model was the main driver of these changes, affecting *CHSY3*, three of the four C4-sulfation isoforms (*CHST11*, *CHST13*, and *CHST14*), and all C6-sulfation isoforms. Remarkably, *CHST15*, which catalyzes 6-sulfation of 4-sulfated *N*-acetylgalactosamine, was modulated under all experimental conditions (**Figure 4D**).

### 3.4 The ocular microbiota model recapitulates most changes induced by individual bacteria and yields a transcriptional profile closer to native corneal tissue

**Figure 5:**
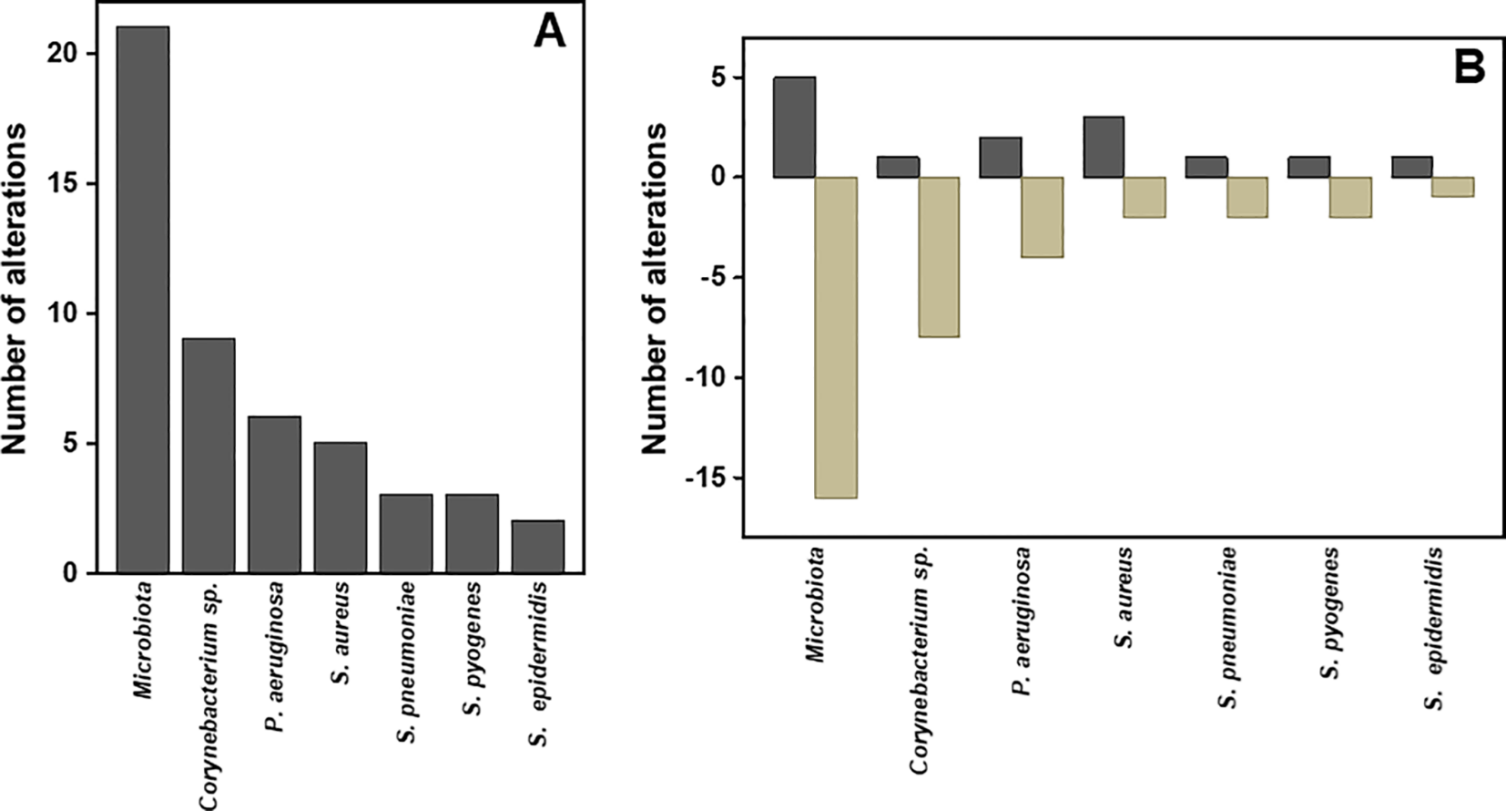
Transcriptional alterations induced by each microorganism and by the microbiota model. **(A)** Total number of genes with altered transcription under each experimental condition. **(B)** Up- and downregulated genes; dark bars indicate the number of genes with increased transcription, whereas light bars indicate the number of genes with decreased transcription.

The number of transcriptional changes varied according to the bacterial species tested. The expression patterns induced by *S. pneumoniae* and *S. pyogenes* were identical, each causing three changes. *S. epidermidis* induced only two modifications, whereas *S. aureus* induced those same two plus three additional ones (five in total). *P. aeruginosa* caused six changes, and *Corynebacterium* sp. nine. In contrast, the microbiota model produced the broadest alteration pattern, affecting 21 genes—five upregulated and 16 downregulated (**Figure 5**).

**Figure 6:**
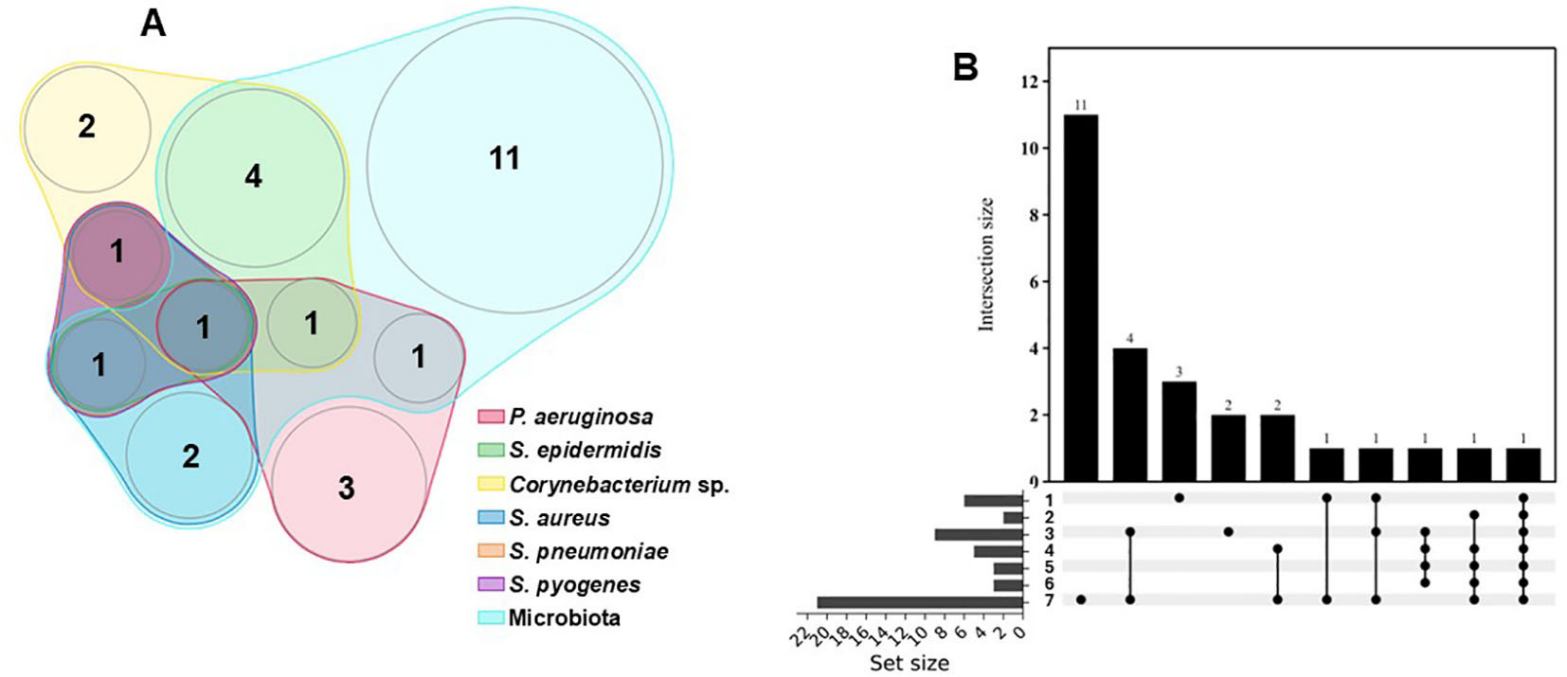
Diagrams showing shared transcriptional alterations among different experimental conditions. **(A)** Venn diagram where colors of each region indicate microorganisms and numbers indicate genes included in each individual or shared region. **(B)** UpSet plot displaying intersections of differentially expressed genes across conditions. The y-axis represents the number of altered genes in each set or intersection, and the x-axis the corresponding condition combinations. Numbers indicate the different treatment conditions: 1, *P. aeruginosa*; 2, *S. epidermidis*; 3, *Corynebacterium* sp.; 4, *S. aureus*; 5, *S. pneumoniae*; 6, *S. pyogenes*; 7, microbiota.

Comparative analysis revealed that the microbiota model encompassed nearly all transcriptional changes observed for individual species, except for three genes exclusively modulated by *P. aeruginosa*, two by *Corynebacterium* sp., and *SRGN*, which was altered by four individual species but not by the microbiota model. In addition, the microbiota induced 11 unique alterations (**Figure 6**).

**Figure 7:**
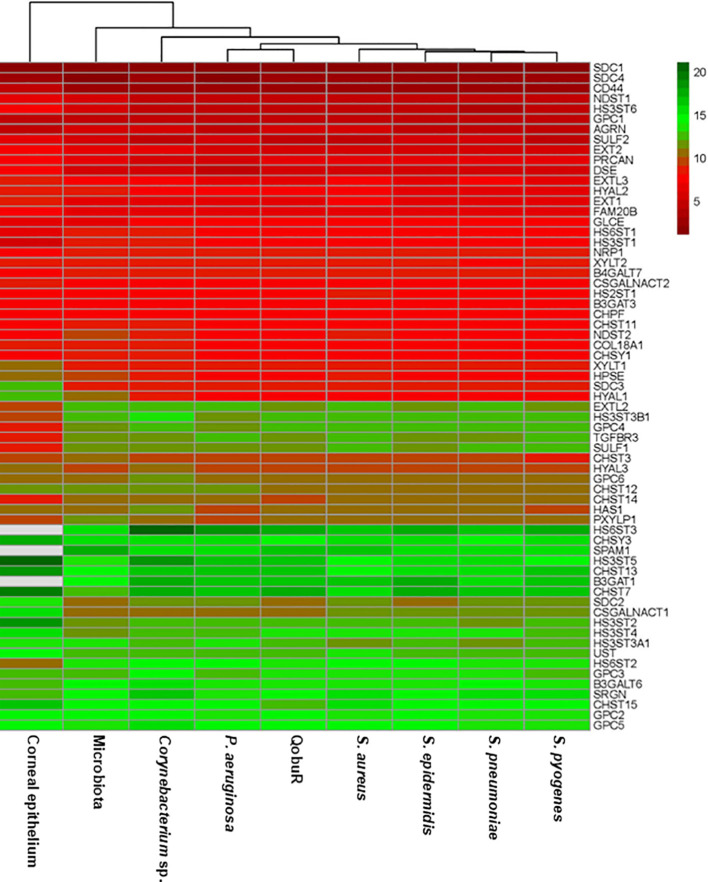
Heatmap and hierarchical clustering of PG- and GAG-related gene transcription across all experimental conditions and donor corneal epithelium. Analyses were performed using normalized qPCR expression values (ΔCt values). Color scale represents normalized ΔCt values.

To evaluate whether gene expression changes induced by microbial exposure, either individual or microbiota-derived, shifted QobuR toward a more native corneal profile, we analyzed transcriptional data from donor-derived corneal epithelia. Hierarchical clustering analysis (**Figure 7**) revealed that the microbiota-treated QobuR appeared closer to donor corneal epithelium, exhibiting the shortest Euclidean distance. In contrast, axenic QobuR showed a divergent expression profile, very similar to that observed after exposure to *P. aeruginosa*. *S. aureus*, *S. pyogenes*, *S. pneumoniae*, and *S. epidermidis* formed a related subgroup, while *Corynebacterium* sp. occupied an intermediate position between these and the microbiota-treated QobuR (**Figure 7**). Hierarchical clustering analyses were based on normalized ΔCt values and therefore reflect global transcriptional similarity patterns among conditions rather than statistical differential expression analyses based on fold changes.

**Figure 8:**
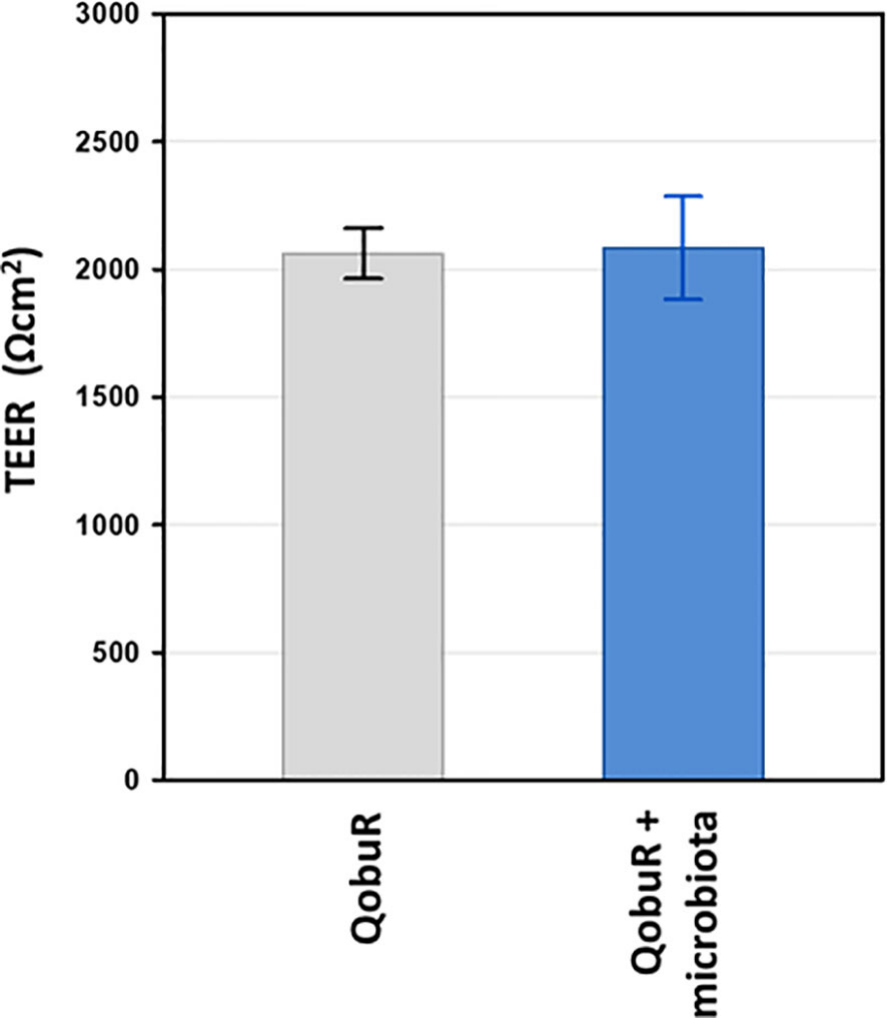
Transepithelial electrical resistance (TEER) measurements of eight reconstructed epithelial models before and after microbiota exposure.

A control experiment was performed to assess whether the epithelial barrier integrity of the CE model was maintained following interaction with microorganisms, by measuring transepithelial electrical resistance (TEER). Incubation of the epithelia with the microbiota model did not alter barrier function, as assessed by TEER, which remained at levels comparable to those observed in the QobuR model prior to treatment (**Figure 8**).

**Figure 9:**
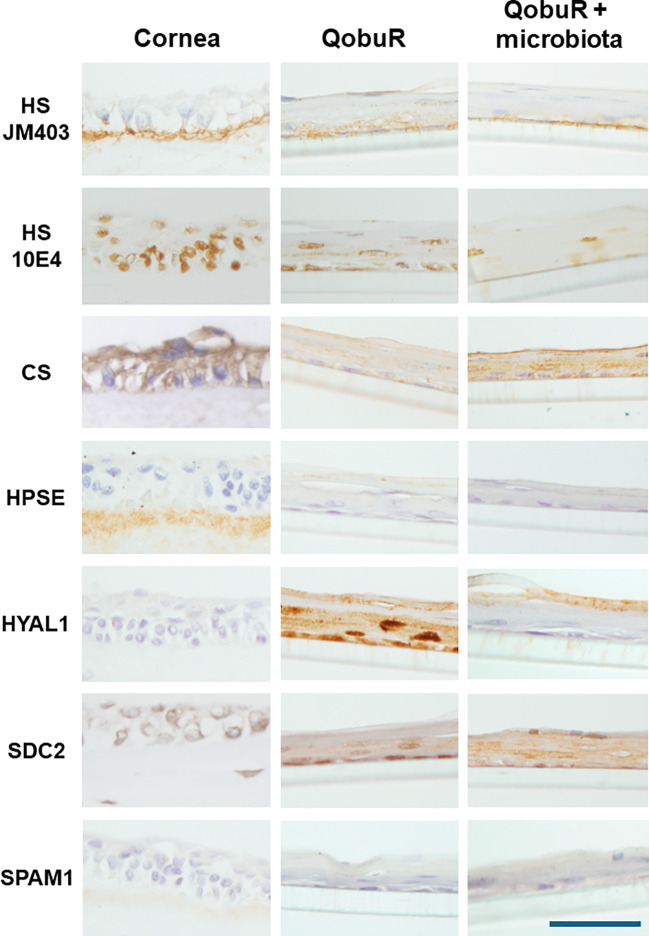
Immunohistochemical analysis of the donor corneal epithelium (left column), QobuR (middle column), and QobuR after exposure to the microbiota model (right column). Staining was performed using anti-HS antibodies recognizing JM403 epitopes (containing N-unsubstituted glucosamine residues) and 10E4 epitopes (containing N-sulfated glucosamine residues), as well as anti-CS antibody (CS-56), anti-heparanase-1, anti-HYAL1, and anti-SPAM1 antibodies. Scale bar: 50 µm. The scale bar is representative of all images shown.

The most relevant alterations detected in QobuR following microbiota exposure were further investigated by immunohistochemistry, with comparison to donor-derived corneal epithelia. HS chains were detected using two antibodies recognizing distinct epitopes, JM403 and 10E4. In donor corneas, anti-JM403 labeling was concentrated at the basal membrane region of the epithelium; in contrast, in axenic QobuR it was more broadly distributed toward the upper epithelial layers. Notably, microbiota exposure resulted in a labeling pattern in which the signal was again concentrated in the basal region (**Figure 9**). Conversely, anti-10E4 staining was predominantly nuclear in both donor corneas and microbiota-treated QobuR, whereas axenic QobuR additionally exhibited some cytoplasmic staining (**Figure 9**).

CS detection revealed strong labeling in donor corneal epithelium, which appeared weaker in the CE model but stronger following microbiota exposure (**Figure 9**). Heparanase was not detected in donor epithelium, although some staining was observed in the proximal stroma. QobuR exhibited weak staining that was no longer detectable upon microbiota exposure (**Figure 9**).

Finally, donor corneal epithelia showed no immunoreactivity for the hyaluronidases HYAL1 or SPAM1. SPAM1 staining in the CE model, with or without microbiota exposure, was also weak. In contrast, HYAL1 staining was evident in axenic QobuR and appeared reduced following microbiota exposure, with only a weak signal remaining in the distal region of the model (**Figure 9**).

## 4 Discussion

The interpretation of these results should be considered within the context of ongoing discussions regarding the existence and functional relevance of a resident corneal microbiota. While increasing evidence supports the presence of microorganisms on the ocular surface, their stability, abundance, and physiological role at the corneal level remain incompletely defined.

The CE constitutes the outermost layer of the cornea, where it performs essential physical and immunological barrier functions. Due to its anatomical location, the CE is exposed to microorganisms present on the ocular surface, and this interaction has been proposed to contribute to ocular homeostasis and the regulation of immune functions (Cavuoto et al., 2019). PGs and GAGs are fundamental molecules in epithelial function, acting as structural scaffolds and regulators of permeability, but also, notably, as modulators of cell signaling (Knupp et al., 2009; Abtahi et al., 2024). This influences processes including proliferation, differentiation, cell migration, and modulation of immune responses (Thoft and Friend, 1983). Furthermore, PGs and GAGs have been implicated in bacterial adhesion to the cornea (Shiju et al., 2020), and it has been reported that their molecular dynamics are altered upon binding to microorganisms, affecting both adhesion and cellular homeostasis (Baker and Chen, 2012).

3D *in vitro* models of the CE are based on the culture of epithelial cells on permeable supports with air–liquid interface exposure, which promotes differentiation and stratification. This yields epithelia that closely resemble native human CE in terms of microstructural morphology, barrier properties, and biomarker expression (Chacón et al., 2019, 2022). However, these epithelia are generated under axenic conditions, making them excellent models for studying the effects of microbial interaction on the expression of PGs and GAGs, their physiological consequences, and the optimization of models relative to native corneas.

Our group previously developed the 3D QobuR model and validated it for preclinical and cellular toxicity studies (Chacón et al., 2019, 2022). Transcriptomic analysis revealed mRNA expression for the vast majority of genes involved in PG and GAG biosynthesis, with only eight exceptions: *B3GAT2*, *EXTL1*, *HAS2–3*, *NDST3–4*, *HPSE2*, and *HYAL4*. *B3GAT2* encodes one of three isoforms involved in the transfer of a glucuronic acid residue as the final step in the synthesis of the tetrasaccharide linker that attaches HS/CS chains to the core protein. It is primarily expressed in the nervous system (Imiya et al., 2002). The other two isoforms, particularly *B3GAT3*, were expressed at high levels. *EXTL1* encodes one of three isoforms involved in HS chain elongation; the other two isoforms were detected, with *EXTL3* notably abundant. *HAS2* and *HAS3* are two of the three hyaluronan synthases found in human cells (DeAngelis and Zimmer, 2023); only *HAS1* was expressed in QobuR. *NDST3* and *NDST4* are two of four NDST isoforms required for HS synthesis, but their expression is restricted to embryonic development (Grobe et al., 2002). *HPSE2* is a homolog of heparanase that lacks HS-degrading activity but can bind HS with high affinity. Finally, *HYAL4* is a CS-specific hydrolase predominantly expressed in the placenta, skeletal muscle, and testis (Kaneiwa et al., 2012). Therefore, QobuR expresses nearly all key genes involved in PG and GAG biosynthesis, making it a suitable model for the precise evaluation of changes under controlled conditions.

In all cases, microbial exposure induced significant remodeling of gene expression, although the effects varied depending on the bacterial species. The tested *Streptococcus* strains produced moderate but consistent effects, impacting two sulfation events on HS and CS chains, specifically via *HS3ST5* and *CHST15*, and the intracellular PG *SRGN*. *Staphylococcus* species elicited a broader range of responses. Two changes (*HS3ST5* and *CHST15*) were shared with *Streptococcus*, but while *S. epidermidis* showed no further changes, *S. aureus* also affected *SRGN* and additional genes involved in CS sulfation. *P. aeruginosa* and *Corynebacterium* sp. differed markedly, exerting the most extensive effects, altering the expression of 6 and 9 genes, respectively, in distinct patterns. Collectively, these findings demonstrate the functional influence of different bacteria on gene expression in the 3D corneal epithelial model and reveal species-specific strategies of epithelial interaction, consistent with previously described mechanisms in the intestinal epithelium (Yang et al., 2025). It should also be noted that the experimental model employed in this study involves a defined co-culture period that may encompass multiple layers of host–microorganism interaction, including not only initial contact but also sustained exposure effects. Therefore, the observed transcriptional changes should be interpreted as reflecting integrated epithelial responses under these controlled conditions, rather than exclusively early adhesion events.

The results obtained with individual bacterial species made it particularly relevant to evaluate the effect of a microbial consortium mimicking the ocular microbiota. Microbiota composition across body sites is highly individual-dependent, as extensively documented for the gut microbiota. In the eye, however, the paucibacterial nature of the ocular surface likely contributes to the substantial variability reported among studies, despite the proposed existence of a relatively stable core microbiota, supported by the recurrent identification of predominant genera in specific proportions. Accordingly, we adopted a model derived from the review by Okonkwo et al., incorporating data from both healthy and pathological conditions, while excluding genera frequently considered potential environmental contaminants such as *Bradyrhizobium*, *Brevundimonas*, and *Aquabacterium* (Okonkwo et al., 2020). The incorporation of additional bacterial species during the development of the microbiota model reflects the iterative nature of the experimental design and the complexity of reproducing microbial exposure conditions representative of the ocular surface. In addition, several studies have reported the presence of potential pathogens as components of the healthy ocular microbiota, ranging from *Staphylococcus epidermidis*, a frequent low-virulence colonizer that contributes to immune homeostasis, to *Pseudomonas aeruginosa*, a more virulent species frequently associated with bacterial keratitis, particularly in contact lens wearers. However, the detection of microorganisms traditionally regarded as pathogens within the healthy ocular surface microbiota should not be interpreted as evidence of infection, but rather as the presence of low-abundance pathobionts whose pathogenic potential depends on epithelial integrity and host–microbe homeostasis (Ozkan and Willcox, 2019).

The use of a microbial consortium designed to approximate the native ocular microbiota produced broader and more complex gene expression alterations than any individual microorganism. A total of 21 genes were affected, including most of the changes observed in single-species exposures. Notably, the microbiota induced 11 unique changes, supporting a synergistic effect exerted by bacterial communities. The absence of these additional effects in individual species highlights the importance of studying microbial interactions as functional consortia.

In a few cases, the transcription of PG core protein genes was affected, and no changes were observed in 14 of the 17 analyzed genes. However, significant downregulation was noted for *SRGN*, with mRNA levels reduced by more than 50% in response to *S. aureus*, *S. pneumoniae*, and *S. pyogenes*, and by up to 80% following exposure to *Corynebacterium* sp. Serglycin is the only known intracellular PG and is preferentially expressed in all inflammatory cells and in tumor cells, although it is also synthesized in non-hematopoietic cell types such as endothelial cells (Kolset and Tveit, 2008; Korpetinou et al., 2014). In the CE model, *SRGN* transcript levels were reduced, being approximately five orders of magnitude lower than those of abundantly expressed PGs such as *SDC1* or *SDC4*. Serglycin is involved in regulating the storage and secretion of inflammatory molecules (Kolset and Tveit, 2008). Its stability in the presence of the microbiota and some commensals (excluding the notable case of *Corynebacterium*) may reflect a state of equilibrium, while its modulation by potential Gram-positive pathogens such as *S. aureus*, *S. pneumoniae*, and *S. pyogenes* suggests a possible immune response adjustment by the epithelium.

Remarkably, most observed changes focused on genes encoding enzymes involved in the biosynthesis of HS and CS, particularly sulfotransferases responsible for fine structural modifications. Regarding HS biosynthesis, *Streptococcus* and *Staphylococcus* species affected only *HS3ST5* transcription. *Corynebacterium* produced broader effects, influencing C6-sulfation of glucosamine by altering expression of isoforms 1 and 3, and C3-sulfation via alternative *HS3ST* isoforms (*-3B1*, *-4*, and *-5*), distinct from those affected by Gram-positive cocci. The most extensive changes were induced by the microbiota, altering N-deacetylation/N-sulfation (via *NDST1-2*) and sulfation at C6 (*HS6ST1*) and C3 (*HS3ST4, -5, -6*) of glucosamine. All changes were restricted to glucosamine sulfation; no alterations were observed in chain polymerization or uronic acid modifications. Importantly, these changes affected both domain-level sulfation (via NDSTs) and terminal C6 and C3 sulfations. The rare C3-sulfation, involving a limited number of residues but essential for specific ligand interactions, was particularly affected, highlighting its biological relevance and the large number of isoforms dedicated to it (Thacker et al., 2014).

Transcription of genes encoding enzymes for CS biosynthesis also exhibited substantial alterations, with 70% of the relevant genes affected. Interestingly, two potential pathogens—*P. aeruginosa* and *S. aureus*—were individually responsible for 4 and 3 gene modifications, respectively. Most changes involved galactosamine sulfation, but effects were also observed in uronic acid epimerization and, notably, in chain polymerization. Again, the microbiota model induced the most widespread changes, affecting one of the three polymerizing isoforms (*CHSY3*), three of four C4-sulfating enzymes (*CHST11, -13, -14*), and all enzymes responsible for C6-sulfation of N-acetylgalactosamine (*CHST3, -7, -15*).

Interestingly, the two genes most affected by different microorganisms, *HS3ST5* and *CHST15*, encode enzymes involved in terminal sulfation events in HS and CS chains, generating highly specialized saccharide units. HS3ST5 is one of seven isoforms catalyzing the rare C3-sulfation of HS glucosamine residues (Whitelock and Iozzo, 2005), a modification associated with Herpes simplex virus infection (Kaneiwa et al., 2012) and underexpression in HeLa cells exposed to microbiota-derived *Lactobacilli* (Martín et al., 2020). *CHST15* encodes an enzyme responsible for C6-sulfation of pre-4-sulfated N-acetylgalactosamine during CS synthesis, generating E and iE disaccharide units (Mikami and Kitagawa, 2013), which have been linked to cell-surface receptor interactions in HSV infections (Mikami and Kitagawa, 2013), chronic inflammation (Begolli et al., 2023), and *Candida albicans* adhesion to skin cells (Ordiales et al., 2022).

Notably, hierarchical cluster analysis comparing QobuR under axenic conditions and after microbial exposure revealed that gene expression patterns in the microbiota-treated epithelium most closely resembled those of ex vivo human corneal epithelium. This suggests that the microbiota-exposed model may more closely reproduce selected features of native corneal epithelium. The inclusion of bacteria in these models may be particularly valuable for studying immune responses, epithelial regeneration, and ocular pharmacodynamics.

The convergence of QobuR transcriptional levels toward those observed in native corneas upon exposure to microbiota models prompted further analysis of the most relevant transcriptional changes relative to axenic cultures and comparison with donor corneas. Variations in mRNA abundance were detected for approximately 30% of the genes involved in HS chain synthesis and 50% of those participating in CS biosynthesis. Additionally, 50% of the genes encoding enzymes responsible for the synthesis of the common tetrasaccharide linker (anchoring the GAG chain to the PG core) were also altered. At present, the mechanisms regulating GAG chain biosynthesis beyond the expression of specific enzymatic isoforms remain poorly understood, making it difficult to establish a direct relationship between these transcriptional alterations and the final structure of the polysaccharide chains (Victor et al., 2009; Huang et al., 2021).

Two antibodies recognizing distinct HS epitopes, 10E4 and JM403, were used in this study. The 10E4 antibody recognizes a native HS epitope containing N-sulfated glucosamine residues (David et al., 1992), whereas JM403 recognizes N-unsubstituted glucosamine residues (van den Born et al., 1995). Anti-10E4 staining produced nuclear labeling in both donor corneas and microbiota-treated QobuR, while axenic QobuR additionally exhibited cytoplasmic staining. In contrast, anti-JM403 labeling was confined to the basal epithelial membrane in both donor corneas and microbiota-treated QobuR, whereas in axenic QobuR it was more diffusely distributed toward the apical epithelial layers.

CS chains were visualized using the CS-56 antibody, which preferentially recognizes CS-D (sulfated at C-2 and C-6) but also detects other structural variants, including CS-A, -B, -C, and -E (Ito et al., 2005). Immunodetection of CS revealed strong labeling in donor corneal epithelium, whereas the CE model displayed weaker staining; notably, microbiota exposure resulted in a staining pattern more closely resembling that of donor epithelium.

Reduced transcriptional levels were also observed for several hydrolases involved in GAG chain degradation. Among these, heparanase-1 and, particularly, HYAL1 showed immunostaining patterns in microbiota-treated QobuR resembling those of native corneal epithelium. HYAL1 staining was evident in axenic cultures but appeared reduced in microbiota-treated conditions, similar to native tissue, where no detectable expression was observed.

Collectively, these findings indicate that both GAG chain composition and the expression of specific enzymatic components in the microbiota-treated CE model more closely resemble those of native CE than those observed under axenic culture conditions.

Furthermore, transepithelial electrical resistance measurements confirmed the preservation of epithelial barrier integrity in the CE model following exposure to the microbiota model. TEER provides a quantitative assessment of epithelial layer integrity and barrier function, with higher values reflecting tighter junctions and enhanced tissue differentiation (Chacón et al., 2019). The observed stability of TEER values indicates that, under the experimental conditions employed, the interaction with the microbiota did not compromise the barrier function of the epithelial models, supporting their suitability for studying host–microbe interactions without disrupting tissue integrity.

Selective modification of GAG chains generates binding sites for a multitude of specific ligands, including cytokines, chemokines, growth factors, enzymes, enzyme inhibitors, and ECM proteins (Whitelock and Iozzo, 2005; Park et al., 2000). To date, 3,464 GAG-binding proteins have been identified, of which 2,873 interact with HS and 720 with CS/DS (Vallet et al., 2022). Through these interactions, GAGs influence numerous physiological and pathological processes, including cell adhesion and migration, ECM organization, regulation of proliferation, differentiation and morphogenesis, cytoskeletal dynamics, tissue repair, inflammation, vascularization, cancer metastasis, infectious pathogenesis, and microbiota interaction (Whitelock and Iozzo, 2005; Martín et al., 2020; Park et al., 2000; García et al., 2016a). Altogether, the data presented in this study support the concept that microbial exposure may modulate epithelial physiology, beyond its traditional role in immune defense, capable of shaping the corneal microenvironment through gene regulation. These findings also establish a foundation for the development of more physiologically relevant 3D CE models for clinical and technological applications.

## Data Availability

The original contributions presented in the study are included in the article/**Supplementary Material**. Further inquiries can be directed to the corresponding authors.
