# Supplementary material for: Microbial modulation of proteoglycan and glycosaminoglycan biosynthesis in a three-dimensional corneal epithelium model
Source: Front Cell Infect Microbiol. 2026 May 28;16:1804227. doi: 10.3389/fcimb.2026.1804227 (PMC13253378; doi:10.3389/fcimb.2026.1804227)
Supplement: Supplementary file 2 [file DataSheet2.pdf]

Supplementary Fig. 1

Differential transcription of genes encoding proteoglycans (PGs) and glycosaminoglycans (GAGs) in axenic and microorganism-treated reconstructed corneal epithelium. Individual graphs show the relative abundance of mRNA transcripts for the different gene groups. Data are displayed on a logarithmic scale for each gene analyzed, and error bars indicate standard deviations. Genes showing statistically significant differences in transcription levels relative to the axenic control are highlighted ($p < 0.05$, Mann–Whitney U test).

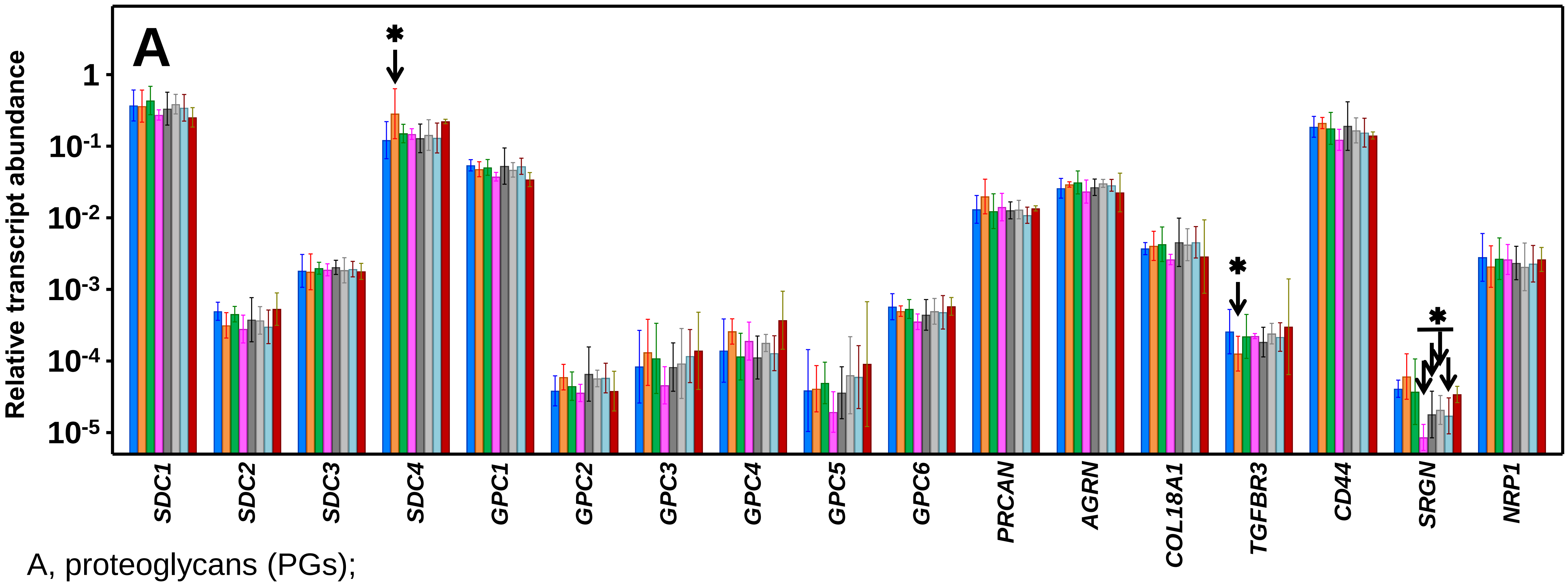

A, proteoglycans (PGs);
From left to right, bars in the graphs represent relative transcript abundance in axenic reconstructed corneal epithelia (dark blue), or after exposure to *Pseudomonas aeruginosa* (orange), *Staphylococcus epidermidis* (green), *Corynebacterium* sp. (purple), *Staphylococcus aureus* (dark gray), *Streptococcus pneumoniae* (light gray), *Streptococcus pyogenes* (light blue), and a microbiota model (dark red).

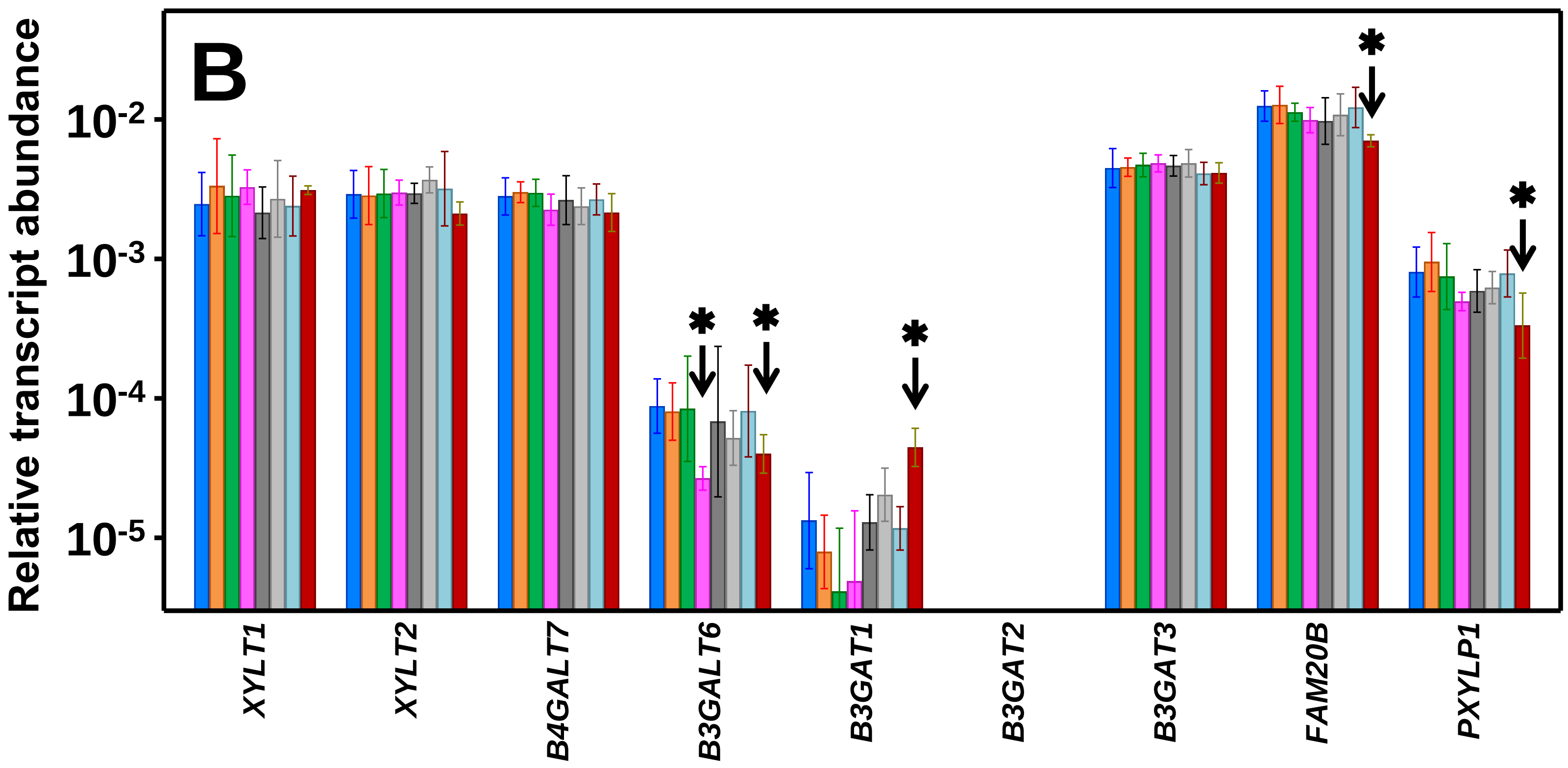

B, the HS/CS tetrasaccharide linker;
From left to right, bars in the graphs represent relative transcript abundance in axenic reconstructed corneal epithelia (dark blue), or after exposure to *Pseudomonas aeruginosa* (orange), *Staphylococcus epidermidis* (green), *Corynebacterium* sp. (purple), *Staphylococcus aureus* (dark gray), *Streptococcus pneumoniae* (light gray), *Streptococcus pyogenes* (light blue), and a microbiota model (dark red).

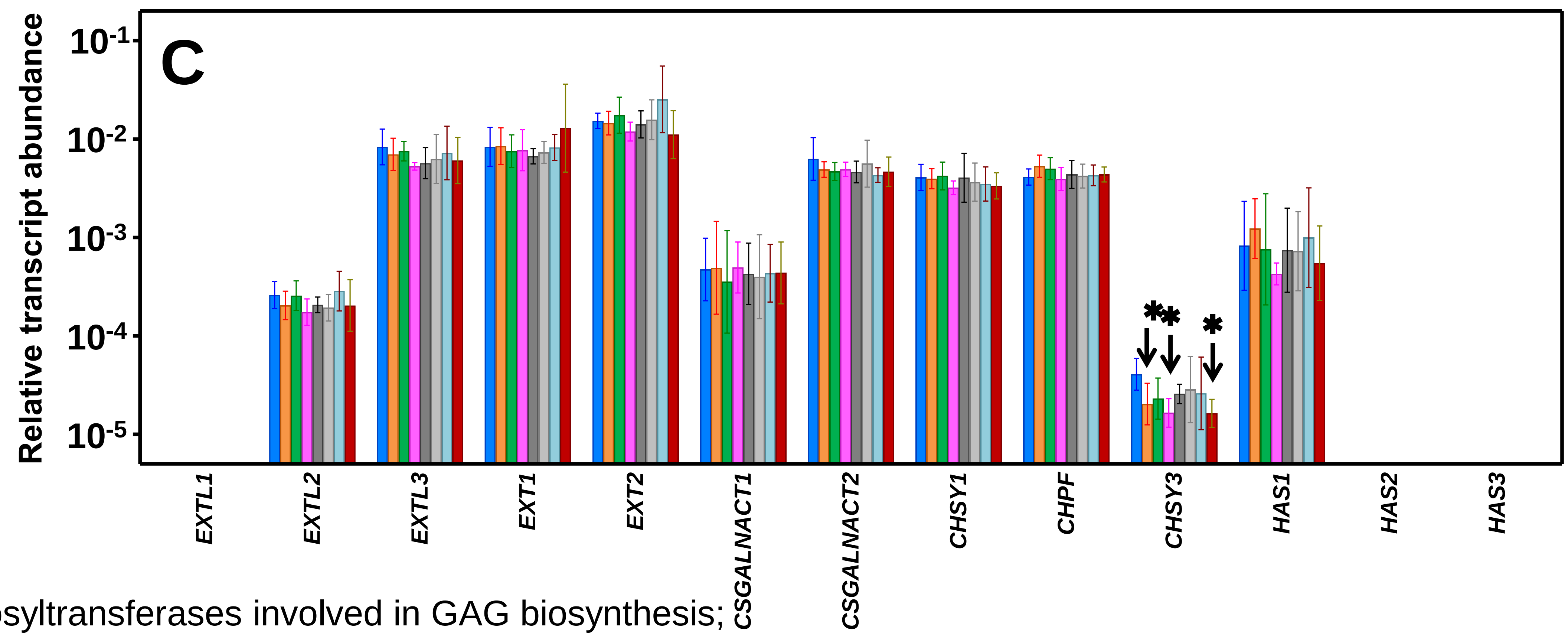

C, glycosyltransferases involved in GAG biosynthesis;
From left to right, bars in the graphs represent relative transcript abundance in axenic reconstructed corneal epithelia (dark blue), or after exposure to *Pseudomonas aeruginosa* (orange), *Staphylococcus epidermidis* (green), *Corynebacterium* sp. (purple), *Staphylococcus aureus* (dark gray), *Streptococcus pneumoniae* (light gray), *Streptococcus pyogenes* (light blue), and a microbiota model (dark red).

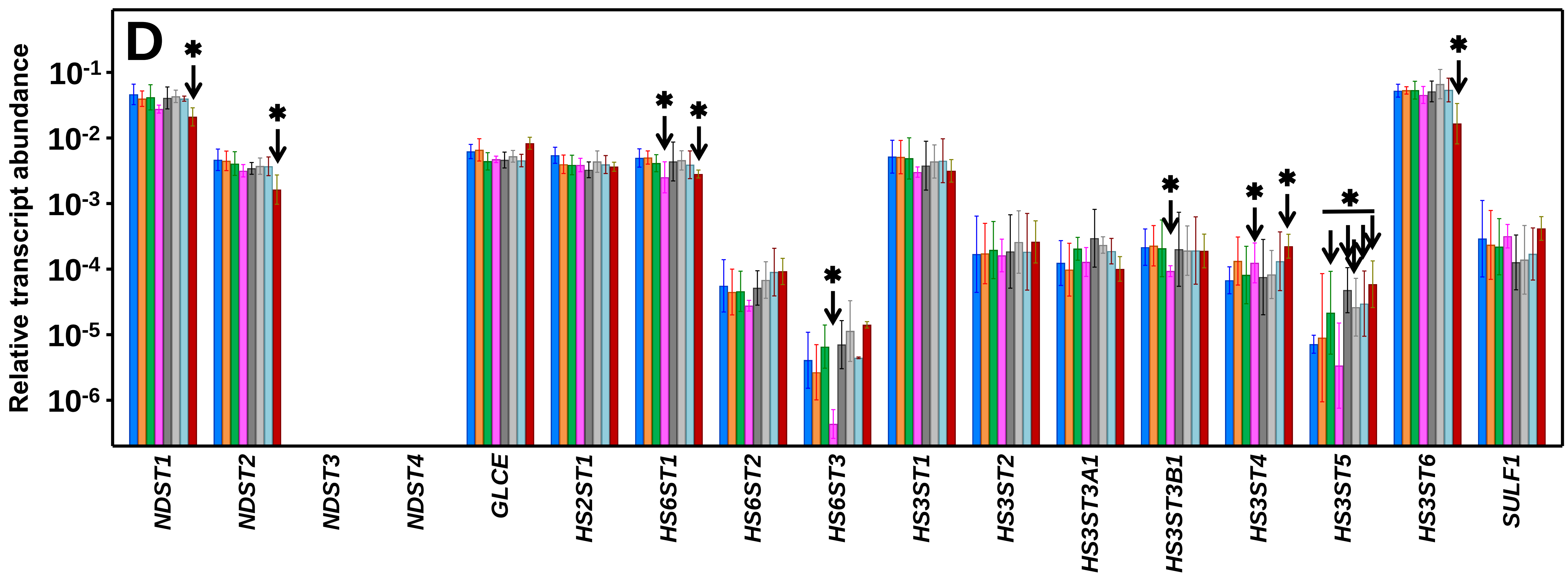

D, genes involved in the structural modification of heparan sulfate (HS);
From left to right, bars in the graphs represent relative transcript abundance in axenic reconstructed corneal epithelia (dark blue), or after exposure to *Pseudomonas aeruginosa* (orange), *Staphylococcus epidermidis* (green), *Corynebacterium* sp. (purple), *Staphylococcus aureus* (dark gray), *Streptococcus pneumoniae* (light gray), *Streptococcus pyogenes* (light blue), and a microbiota model (dark red).

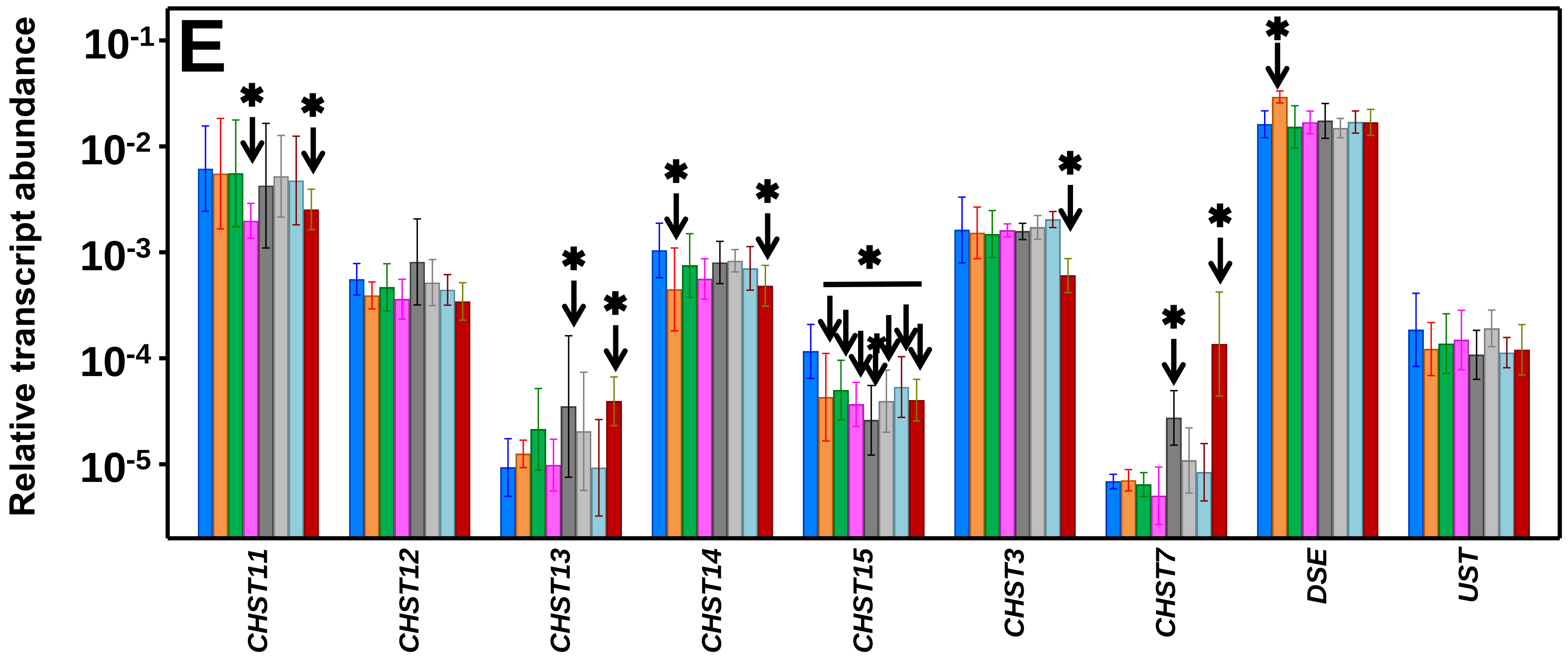

E, genes involved in the structural modification of chondroitin sulfate (CS);
From left to right, bars in the graphs represent relative transcript abundance in axenic reconstructed corneal epithelia (dark blue), or after exposure to *Pseudomonas aeruginosa* (orange), *Staphylococcus epidermidis* (green), *Corynebacterium* sp. (purple), *Staphylococcus aureus* (dark gray), *Streptococcus pneumoniae* (light gray), *Streptococcus pyogenes* (light blue), and a microbiota model (dark red).

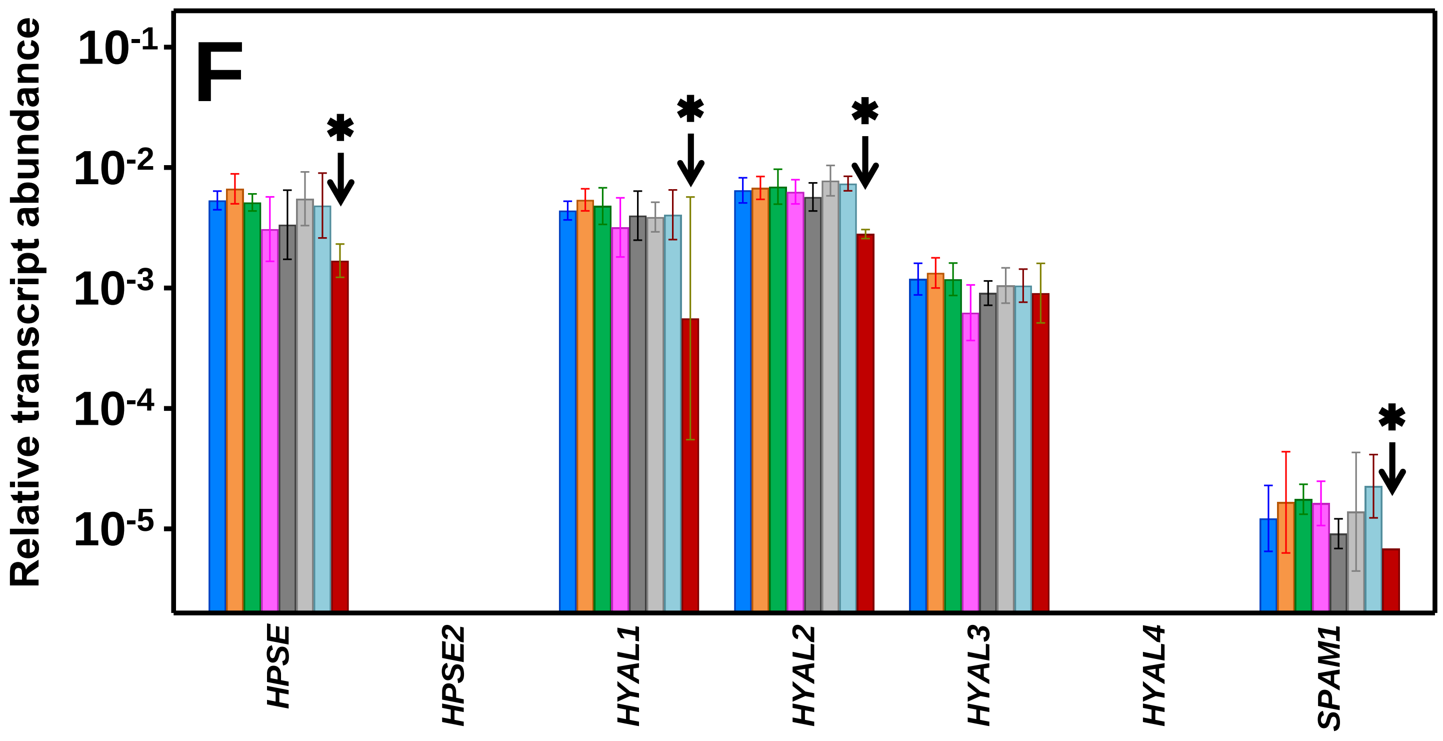

F, GAG-degrading glycosyl hydrolases.
From left to right, bars in the graphs represent relative transcript abundance in axenic reconstructed corneal epithelia (dark blue), or after exposure to *Pseudomonas aeruginosa* (orange), *Staphylococcus epidermidis* (green), *Corynebacterium* sp. (purple), *Staphylococcus aureus* (dark gray), *Streptococcus pneumoniae* (light gray), *Streptococcus pyogenes* (light blue), and a microbiota model (dark red).
